# Evidence of anthropogenic impacts on global drought frequency, duration, and intensity

**DOI:** 10.1038/s41467-021-22314-w

**Published:** 2021-05-12

**Authors:** Felicia Chiang, Omid Mazdiyasni, Amir AghaKouchak

**Affiliations:** 1 Department of Civil and Environmental Engineering, University of California, Irvine, CA USA; 2 Department of Earth System Science, University of California, Irvine, CA USA

**Keywords:** Attribution, Hydrology

## Abstract

Most climate change detection and attribution studies have focused on mean or extreme temperature or precipitation, neglecting to explore long-term changes in drought characteristics. Here we provide evidence that anthropogenic forcing has impacted interrelated meteorological drought characteristics. Using SPI and SPEI indices generated from an ensemble of 9 CMIP6 models (using 3 realizations per model), we show that the presence of anthropogenic forcing has increased the drought frequency, maximum drought duration, and maximum drought intensity experienced in large parts of the Americas, Africa, and Asia. Using individual greenhouse gas and anthropogenic aerosol forcings, we also highlight that regional balances between the two major forcings have contributed to the drying patterns detected in our results. Overall, we provide a comprehensive characterization of the influence of anthropogenic forcing on drought characteristics, providing important perspectives on the role of forcings in driving changes in drought events.

## Abstract (web summary)

Most studies have examined the impacts of human-driven climate change on mean or extreme climate variables and have neglected to explore interrelated drought features. Here, the authors show that the presence of human activity has increased the number and maximum length and intensity of drought events across the globe.

## Introduction

Droughts have severe direct and indirect impacts on ecological, agricultural, and economic sectors, such as damage to wildlife habitats and crops^1–5^. Relatively low water availability (i.e., droughts) can also have strong ramifications on solar thermal, geothermal, and hydropower generation^6,7^. In addition, drought events can influence the occurrence of dependent hazards, such as heatwaves, and the concurrence of such events can ‘cascade’ to increase the risk of wildfire events^8,9^. Global Precipitation Climatology Center (GPCC) observations have shown positive trends in meteorological drought frequency, duration, and intensity in Western Africa, East Asia, Central America, the Amazon, and the Mediterranean between 1951 and 2010^10^. In addition, Climatic Research Unit (CRU) observations have also shown a significant positive trend in land areas under meteorological drought^11^. Since future climate projections suggest increases in drought frequency and severity in the Americas, Europe, Asia, and Africa, the characterization of drought features is an important and relevant area of study in the field of hydrology^12–17^.

Previous detection and attribution studies have used observations and model simulations to attribute increasing trends of mean and extreme temperature and precipitation occurrences to anthropogenic emissions^18–23^. Many detection and attribution studies have used model simulations of our historical climate with and without anthropogenic forcing to determine whether our observed climate conditions are due to natural variability, anthropogenic emissions, or a combination of the two^19,24^. Using a fractional risk measure (referred to as ‘fraction of attributable risk’), Fischer and Knutti^18^ found that 18% of moderate daily precipitation extremes can be attributed to the present-day 0.85 °C temperature increase. In addition, Fischer and Knutti attributed 75% of the moderate daily hot extremes to the ongoing increase in global temperatures^18^.

Although there have been many detection and attribution studies on hydroclimatic variables in the literature^18,19,22,24–26^, the global influence of anthropogenic forcing on different drought characteristics (e.g., duration, frequency, severity) has not been explicitly quantified. Previously, Wehner et al. reviewed the changes that have occurred in different drought types in the United States^27^. So far, historical changes in meteorological drought conditions in the U.S. have not been formally attributed to anthropogenic forcing^27^. On the other hand, hydrological drought conditions in the Western U.S. have been attributed to anthropogenic forcing, since they are dependent on snowfall accumulation and eventual snowmelt, which are strongly influenced by temperature conditions^25^. In addition, Marvel et al. used climate model simulations and drought atlases constructed from tree ring records to present evidence of the influence of human activity on global soil moisture drought trends since the start of the 20^th^ century^28^. Marvel et al. detected an overall increasing signal of human activity in the global drought atlas region, with a decreasing signal during 1950–1975 that may have resulted from anthropogenic aerosol emissions during the time period. Bonfils et al. also recently used a multivariate fingerprinting approach to capture the contribution of human emissions on drying patterns across the globe, demonstrating that observed changes in hydroclimatic conditions stem from the combination of greenhouse gases and anthropogenic and natural aerosol forcings^29^. On the whole, previous studies have shown that the influence of anthropogenic forcing on drought events is complicated, due to the variety of ways that drought can be characterized.

In this work, we examine the influence of anthropogenic forcing on changes in meteorological drought characteristics across the globe. Here we use the recently released Coupled Model Intercomparison Project Phase 6 (CMIP6) climate model simulations to create standardized precipitation indices (SPI) to characterize meteorological droughts. Using 9 CMIP6 models (with 3 ensemble members per model), we quantified the drought frequency, maximum drought duration, and maximum drought intensity of historical (including all greenhouse-gas (GHG) and anthropogenic aerosol (AER) forcings) and historical natural-only climate scenarios to examine the impact of anthropogenic emissions on shifts in drought features between the 19^th^ and the 20^th^ centuries. In addition, we used a spatially aggregated perspective to evaluate the impact of anthropogenic forcing on the global distributions of shifts in these drought features. We also examined differences in bivariate distributions of drought event duration and median intensity to characterize the impact of anthropogenic forcing on the dependence between these drought features.

To directly quantify the impact of anthropogenic forcing on drought events, we estimated the likelihood of drought occurrences that can be attributed to anthropogenic climate change. We employed the probability ratio concept used in Fischer and Knutti (2015) to quantify the likelihood of drought events occurring in historical conditions relative to natural-only conditions in the late 20^th^ century^18^. By examining the global distribution of drought risk, we identified regions of greater sensitivity to anthropogenic climate change from a meteorological drought perspective. We additionally analyzed the individual impacts of GHG-only and AER-only forcings on the likelihood of drought occurrences to gain a better understanding of the individual contributions of greenhouse gases and anthropogenic aerosol forcings. In addition to our SPI-based analysis, we examined drought characteristics based on standardized precipitation-evapotranspiration index (SPEI) data to better understand anthropogenic forcing impacts on net water availability, regarded here as the difference between monthly precipitation and potential evapotranspiration^30^. By using SPEI, we could examine the additional impact of anthropogenic forcing on the evaporative demand of the atmosphere, which is expected to increase as global temperatures continue to rise^31^. Overall, understanding the contribution of anthropogenic climate change to drought characteristics is important in our interpretation of historically observed drought trends.

## Results

**Fig. 1:**
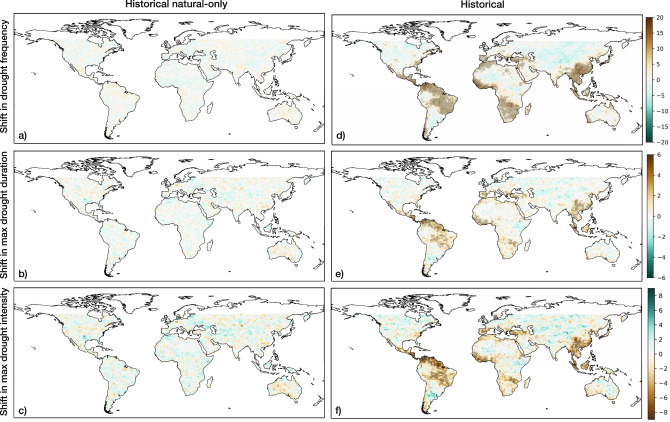
Global shifts in 6-month SPI drought features from historical and historical natural-only simulations with a defined drought threshold of SPI < −1.5. Stippling over each pixel indicates a statistically significant increase in the associated drought characteristic (see Methods). **a** Difference in drought frequency between 1956–2005 and 1851–1900 from the CMIP6 historical natural-only multi-model ensemble median. **b** Difference in maximum drought duration under historical natural-only conditions. **c** Difference in maximum drought intensity under historical natural-only conditions. **d** Difference in drought frequency under historical conditions. **e** Difference in historical maximum drought duration. **f** Difference in historical maximum drought intensity.

Here, we first examine how SPI drought frequency, duration, and intensity have evolved over our study period in historical and historical natural-only conditions. Figure 1 depicts global patterns of multi-model median shifts in drought frequency, maximum drought duration, and maximum drought intensity between 1851–1900 and 1956–2005. Figure 1a, b, and c, which represents changes in (a) drought frequency, (b) maximum drought duration, and (c) maximum drought intensity under historical natural-only conditions, does not show any coherent regional changes from the late 19th to the late 20th centuries. From a global perspective, the three features have shifted towards a slightly wetter profile. In contrast to the historical natural-only shifts, Fig. 1d shows statistically significant hotspots of change in drought occurrences in southern Europe, Central and South America, western and southern Africa, and eastern Asia under historical conditions. In addition, Fig. 1e, f shows similar substantial regional changes in maximum drought duration and intensity. In general, the modeled changes in drought characteristics between the two time periods reflect trends in observed drought frequency, duration, and severity that have been documented in Spinoni et al.^10^. From Fig. 1, we observe that many of the regions where drought features have developed a stronger presence in historical conditions are not necessarily regions where drought features experienced increases as a result of natural climate variations. This highlights the importance of accurately capturing natural variability in the models to understand the contribution of present-day and future anthropogenic climate change to drought features.

**Fig. 2:**
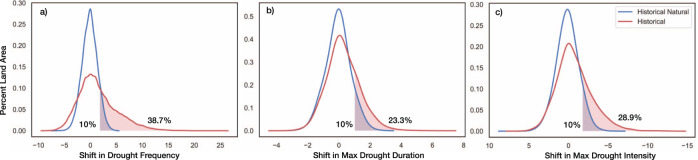
Global land distributions of 6-month SPI drought feature shifts from historical and historical natural simulations. Each probability density function represents the distribution of shifts between 1956–2005 and 1851–1900 in **a** drought frequency, **b** maximum drought duration, **c** maximum drought intensity from all land pixels between 60°N and 60°S.

To globally summarize the changes that have occurred in historical and historical natural-only simulations, we also generated spatially aggregated distributions of each SPI-based drought characteristic from Fig. 1 (see Supplementary Table 1 for two-tailed two-sample *t*-test results). Figure 2 displays spatial probability distributions constructed using all land pixels between 60°N and 60°S. From Fig. 2, we can see strong upper tail differences in all three distributions, indicating greater increases in drought frequency, duration, and intensity under historical conditions relative to historical natural-only conditions. The distributions of the drought characteristics under historical conditions also possess much greater variability relative to the distributions driven solely by internal climate variability. The differences between historical and historical natural-only conditions signify that significant increases in the number of drought occurrences, the length of the maximum drought duration, and the magnitude of the maximum drought intensity have occurred due to the presence of anthropogenic forcing from a globally aggregated perspective.

**Fig. 3:**
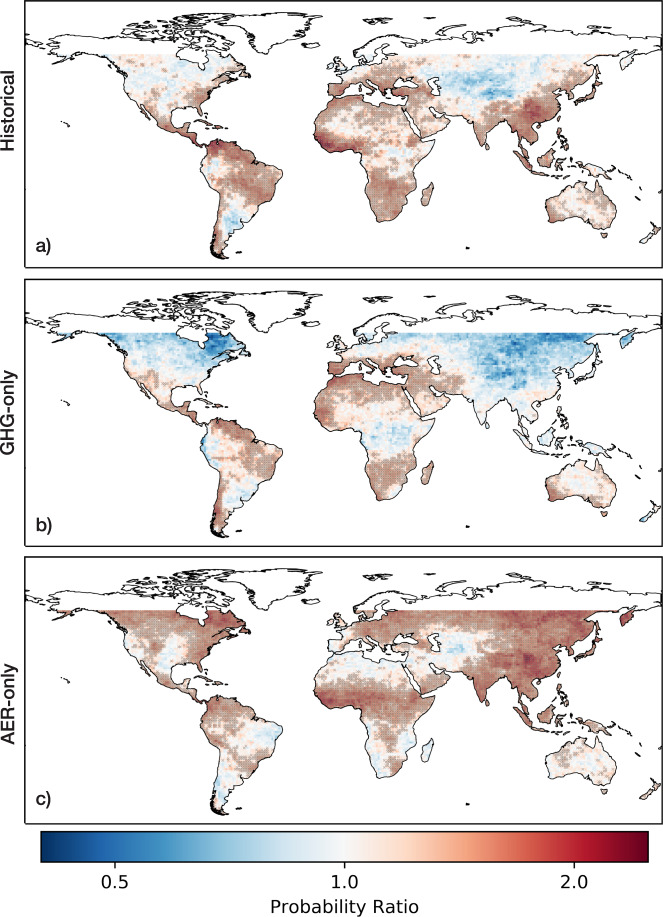
Probability Ratios (PR) of 6-month SPI drought events under historical, greenhouse gas (GHG-only), and aerosol (AER-only) forcings. The PR of each pixel is calculated by designating 6-month SPI dips less than −1.5 as drought events, using SPI data generated from **a** historical, **b** GHG-only, and **c** AER-only forced CMIP6 models. Values above 1 indicate higher risks of drought events in forced conditions, while values below 1 indicate lower risks of drought events in natural-only conditions. Stippling over the grid cells indicates that the median of the model ensemble is statistically significantly greater than 1.

To better comprehend the influence of anthropogenic forcing on SPI drought occurrences, we visualize the probability ratio (PR) of drought events occurring under historical over historical natural-only conditions during 1956–2005 (Fig. 3a). Using a 6-month SPI window, we calculated PR values for each land pixel to understand climate change impacts on the frequency of drought events (see Supplementary Figs. 1 and 2 for alternative SPI thresholds and windows). The resulting global PR pattern shown in Fig. 3a generally reflects the spatial shifts in drought frequency shown in Fig. 1. However, by directly comparing the likelihood of drought under historical over historical natural-only conditions, we see that parts of southern and eastern Europe, northern and western Africa, and India experience significant divergences that are not immediately apparent when only examining historical trends and shifts.

In Fig. 3b, c, we examine the impact of individual GHG-only and AER-only forcings on the probability ratios of 6-month SPI drought occurrences during 1956–2005 (see Supplementary Fig. 3 for shifts in drought features). By comparing GHG-only and historical natural-only climate conditions, we show that the presence of greenhouse gases has played a significant role in increasing the likelihood of drought occurrences in southern Europe, northern and southern Africa, Central America, and parts of South America (Fig. 3b). In addition, we see that the spatial distribution of the probability ratios of drought occurrences corresponds well with 21^st^ century multi-model projections of changes in mean precipitation^32,33^. Therefore, climate models suggest that the forced response to greenhouse gas emissions will persist and dominate in the 21^st^ century. These large-scale changes in meteorological droughts can be explained by thermodynamic and dynamic responses to the presence of greenhouse gases^32,34–36^. From a thermodynamic perspective, atmospheric water vapor increases alongside warming temperatures, intensifying the transportation of water vapor and resulting in wet regions getting wetter and dry regions getting drier^34–36^. We see this expressed in the wetting of the mid and high latitude regions and the drying of the subtropical dry regions^32^. However, it is important to note that this thermodynamic response does not hold for all land areas, such as the Amazon^32^. From a dynamic perspective, greenhouse gas emissions produce changes in atmospheric circulation patterns, and we see this manifest in the poleward expansion of the Hadley cell and the poleward expansion of subtropical dry regions^32,36^.

The forced response to anthropogenic aerosols produces a very different global pattern of drought probability ratios. Anthropogenic aerosols play a significant role in South and East Asia, in addition to Central America, the Sahel region of Africa, and the higher latitudes bordering the sub-arctic regions (Fig. 3c). Our results correspond well with previous studies examining anthropogenic-aerosol-forced changes in precipitation^37,38^. Previous regional studies identified aerosol emissions as a major contributor to decreases in precipitation in Northern Hemisphere monsoon regions^39–44^. For example, Biasutti and Giannini (2006) demonstrated that late 20^th^ century drying in the Sahel region was influenced by the presence of anthropogenic emissions^42^. By comparing simulations forced by aerosols and greenhouse gases with simulations forced by greenhouse gases alone, Biasutti and Giannini presented evidence that reflective aerosols played a key role in altering Atlantic sea surface temperature patterns, which induced the observed drying in the Sahel^42^. In addition, Bollasina et al.^41^ showed that anthropogenic aerosol emissions played a major role in decreasing land precipitation generally expected from the South Asian summer monsoon^41^. Bollasina et al. argued that the presence of local aerosols decreases the local thermal contrast between the land and sea surfaces and reduces the meridional air temperature and sea-level pressure gradients, thus weakening the South Asian monsoon^41^.

By examining the individually forced responses to greenhouse gases and to anthropogenic aerosols, we illustrate how historical patterns of changes in drought occurrences are dependent on regional balances between greenhouse gases and aerosols. We also show that future projections of changes in drought occurrences are already expressed in historical responses to the individual presence of greenhouse gases. The drought response in Asian and African monsoonal regions is more clearly attributed to anthropogenic aerosols and thus, we can expect that these specific regional changes in drought occurrences will not persist as aerosol emissions decline.

Using regions delineated by the Intergovernmental Panel on Climate Change AR5 report (hereafter referred to as IPCC regions), we also studied whether the dependence structure between drought duration and intensity differs under historical and historical natural-only conditions^32^. To examine this, for each region, we generated bivariate kernel density estimates constructed from the duration and median intensity of each non-consecutive drought event falling below a 6-month SPI threshold of −1.5 between 1850–2005 from all CMIP6 models. In addition, we tested whether historical and historical natural-only distributions were statistically different for each IPCC region with Fasano and Franceschini’s 2-dimensional, 2-sample Kolmogorov–Smirnov test^45,46^. We found that most regions exhibited statistical significance at an alpha level of 0.05, except for the Tibetan Plateau (see Supplementary Table 2).

**Fig. 4:**
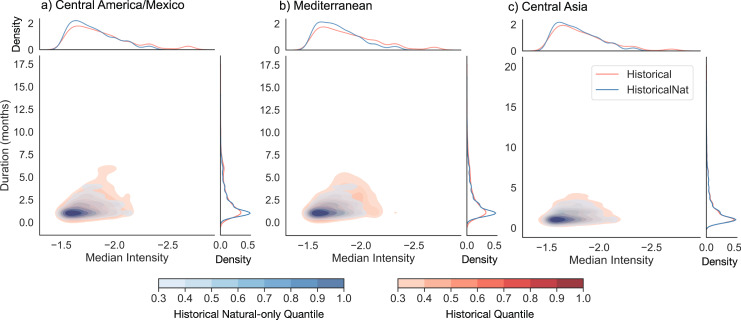
Bivariate distributions of drought event duration and median intensity. Each distribution is based on 6-month SPI < −1.5 droughts identified from **a**) Central America/Mexico, **b**) Mediterranean, and **c**) Central Asia regional data from all CMIP6 models. Univariate distributions of each characteristic are displayed on the outer axes of each subplot.

Figure 4 presents the distributions from three of the IPCC regions: Central America/Mexico, the Mediterranean, and Central Asia (see Supplementary Fig. 4 for the remaining regions). For Central America/Mexico and the Mediterranean, we see concurrent shifts in event duration and median intensity from historical natural-only conditions to historical conditions, with substantial increases found at the tail of the median intensity distribution. Increases at the tail of the median intensity distribution are also found in Central Asia and many of the other IPCC regions (see Supplementary Fig. 4). These tail increases signify that many regions are experiencing levels of drought intensity not previously seen in pre-industrial conditions. Broadly, these results signify that although many regions, such as Central Asia, have not experienced pronounced changes in individual drought features (as seen in Fig. 1), historical distributions of drought event duration and intensity already significantly differ from distributions constructed from natural-only conditions. More in-depth analyses on the nature of joint changes in drought duration and intensity would provide important multivariate perspectives on changes in meteorological drought characteristics forced by anthropogenic emissions.

**Fig. 5:**
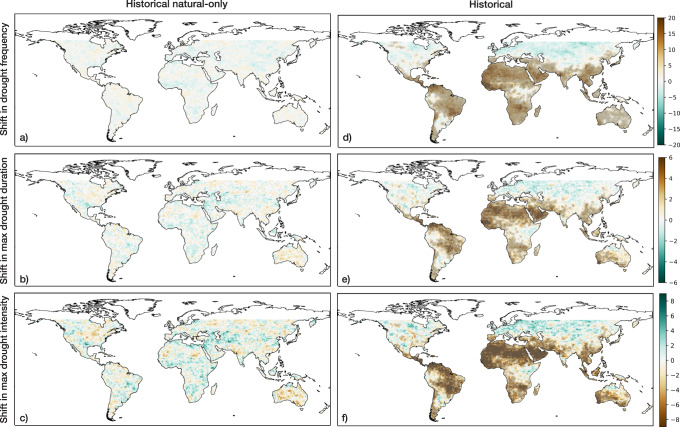
Global shifts in SPEI drought features from historical and historical natural-only simulations. Stippling over each pixel indicates a statistically significant increase in the associated drought feature (see Methods). **a** Difference in drought frequency between 1956–2005 and 1851–1900 from the CMIP6 historical natural-only multi-model ensemble median. **b** Difference in maximum drought duration under historical natural-only conditions. **c** Difference in maximum drought intensity under historical natural-only conditions. **d** Difference in drought frequency under historical conditions. **e** Difference in historical maximum drought duration. **f** Difference in historical maximum drought intensity.

To examine the impact of anthropogenic forcing on net water availability, we also characterized droughts from standardized precipitation-evapotranspiration index (SPEI) data. With SPEI, we account for atmospheric evaporative demand, represented as potential evapotranspiration, in our forced response. Figure 5 depicts shifts in SPEI drought frequency, maximum drought duration, and maximum drought intensity under historical and historical natural-only conditions. Figure 5a, b, and c, which displays changes in drought frequency, maximum drought duration, and maximum drought intensity under historical natural-only conditions, does not show distinctive regional patterns between the late 19^th^ and 20^th^ centuries. Similar to Fig. 1, the SPEI-based historical natural-only plots exhibit subtle worldwide decreases in all drought characteristics. In addition, similar to the historical SPI results shown in Fig. 1d, Fig. 5d also shows strong statistically significant shifts in historical drought frequency between the late 19^th^ and 20^th^ centuries. Figure 5e, f, which depicts historical maximum drought duration and maximum drought intensity, also corresponds well with the spatial changes depicted in historical drought frequency.

**Fig. 6:**
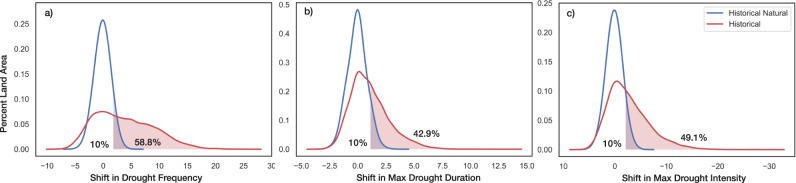
Global land distributions of SPEI-based drought feature shifts from historical and historical natural simulations. Each probability density function represents the distribution of shifts between 1956–2005 and 1851–1900 in **a** drought frequency, **b** maximum drought duration, **c** maximum drought intensity from all land pixels between 60°N and 60°S.

We also generated spatially aggregated distributions of each SPEI-based drought characteristic from Fig. 5 (see Supplementary Table 3 for two-tailed two-sample *t*-test results). In Fig. 6, when we compare the shifts that have occurred under historical and historical natural-only climate conditions, we also see strong upper tail divergences between the SPEI-based distributions, mirroring the SPI-based distributions depicted in Fig. 2. This indicates that strong increases in drought frequency, duration, and intensity have also occurred due to anthropogenic forcing when evaluating meteorological net water availability.

**Fig. 7:**
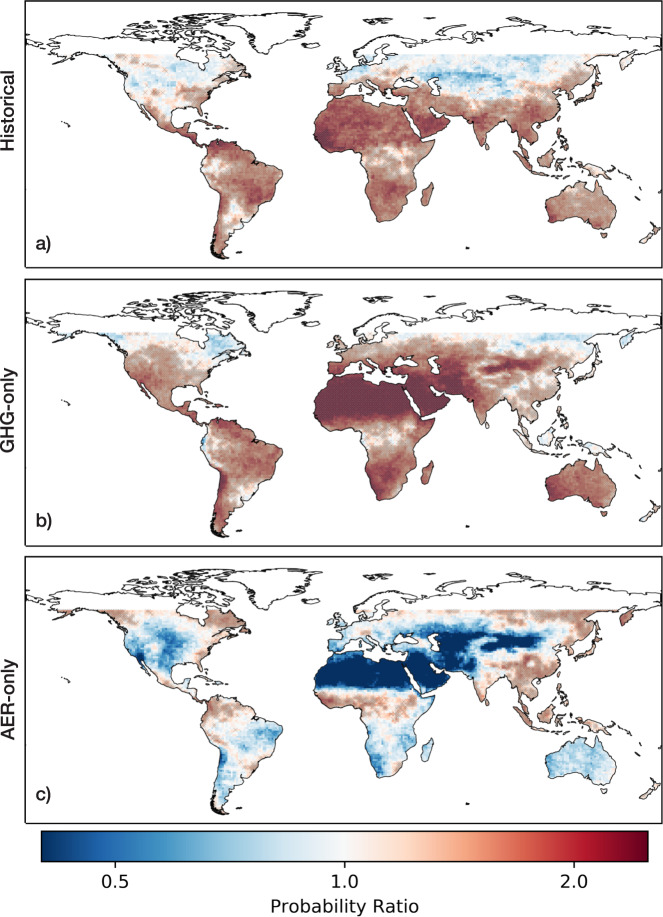
SPEI-based Probability Ratio (PR) plots for historical, greenhouse gas (GHG-only), and aerosol (AER-only) conditions. The PR of each pixel is calculated by designating 6-month SPEI dips less than -1.5 as drought events, using **a** historical, **b** GHG-only, and **c** AER-only datasets. Values above 1 indicate higher risks of drought events in forced conditions, while values below 1 indicate lower risks of drought events in natural-only conditions. Stippling over the grid cells indicates that the median of the model ensemble is statistically significantly greater than 1.

Finally, we examined historical, GHG-only, and AER-only probability ratios based on SPEI drought occurrences during the period 1956–2005 to provide perspective on the added impact of anthropogenic forcing on evaporative demand (Fig. 7). When examining historical conditions, compared to our SPI-based probability ratios, we observe much greater increases in SPEI-based drought probability ratios, especially in the tropical and subtropical latitude bands (Fig. 7a). This falls in line with previous literature regarding observed and expected changes in potential evapotranspiration in response to anthropogenic climate change. Sherwood and Fu (2014) previously highlighted that potential evapotranspiration over land is expected to increase dramatically in most tropical and subtropical regions in response to warming temperatures, shifting local climates to adopt drier background states^31^. Naturally, drier climates would be more likely to experience historical natural-only defined drought events, which supports our findings. When examining the individual influence of greenhouse gases on net water availability, we see an even greater increase in SPEI-based drought probability ratios across the globe (Fig. 7b). This demonstrates the distinct response of potential evapotranspiration to the presence of greenhouse gas forcing. In contrast, when we focus on the individual influence of aerosols on net water availability, we see strong decreases in the probability ratios over the tropical and subtropical bands which is likely due to aerosol driven decreases in potential evapotranspiration (Fig. 7c). However, as we can see in Fig. 7a, the greenhouse gas forcing overwhelmingly dominates over the aerosol forcing across much of the globe, indicating the strong influence of greenhouse gases on potential evapotranspiration, and consequently, net water availability.

## Discussion

Using CMIP6 model simulations, we provide a spatial, model-based meteorological perspective regarding which regions have experienced greater changes in drought characteristics due to anthropogenic forcing between the late 19^th^ and 20^th^ centuries. From a globally aggregated perspective, we show that SPI-based drought frequency, duration, and intensity distributions have significantly shifted due to anthropogenic forcing. We specifically show that the presence of anthropogenic forcing has increased the frequency, duration, and intensity of SPI-based droughts, specifically in the Americas, the Mediterranean, western and southern Africa, and eastern Asia. When we examine greenhouse gases and anthropogenic aerosol forcings separately, we find that greenhouse gases have significantly influenced drought occurrences in the Mediterranean, Central America, the Amazon, and southern Africa, while anthropogenic aerosols have played a larger role in Northern Hemisphere monsoonal and sub-arctic regions. In addition, the inclusion of atmospheric demand in our drought definition significantly increases the global likelihood of drought occurrences due to the overwhelming influence of greenhouse gases on potential evapotranspiration.

We acknowledge there are limitations associated with our model-based detection study. Drying trends from Nasrollahi et al. showed that the majority of CMIP5 models mirror trends of areas under drought observed from Climatic Research Unit (CRU) data^11^. However, there are still regional disparities that exist between observations and model simulations regarding drying and wetting trends^11^. CMIP6 (and previous CMIP5) models suffer from tropical sea surface temperature (SST) biases, which can impact the accuracy of El Niño-Southern Oscillation (ENSO) simulations^47–49^. These SST biases can affect the simulation of droughts in regions that are strongly teleconnected to ENSO events^47^. In addition, the double-intertropical convergence zone (ITCZ) is still present in the CMIP6 models, which contributes to precipitation biases^50^. However, we highlight that the general pattern of ENSO variability and associated precipitation teleconnections are reasonably represented in the models^51^.

As we still lack a good understanding of aerosol feedbacks, CMIP6 models may not able to comprehensively replicate real-world changes in drought features resulting from anthropogenic aerosol emissions^28^. In addition, models may not fully capture the scope of vegetation responses to increasing levels of CO_2_ and rising temperatures; these responses have been shown to influence regional hydroclimatic conditions^52,53^. Previous studies have also shown that biases and uncertainties in model simulations can influence the detection of climate change signals and the magnitude of drought trends^54,55^. Due to these model biases, we acknowledge that there is a degree of error with regard to our results. However, since this study examined differences between model experiments, the significance of these model biases on our results is reduced. As we examined temporal shifts between large time periods, we also minimized the influence of short-term interdecadal internal variability on our results. We also highlight that future studies focusing on different types of droughts (e.g., hydrological, agricultural) may reveal regional differences due to varying responses to anthropogenic forcings^56,57^. With regard to our SPEI results, we acknowledge the sensitivity of potential ET to temperature and further evaluation of other supply and demand indicators may provide useful perspectives regarding the impacts of human-driven climate change on drought events.

In general, anthropogenic forcing has played a significant role in increasing meteorological drought frequency, duration, and intensity in many regions across the globe. Regional balances between greenhouse gases and anthropogenic aerosols have contributed to the wetting and drying patterns found in our results. However, current climate models indicate that the individual greenhouse gas signal found in historical model simulations is expected to persist and dominate over anthropogenic aerosols in the 21^st^ century. We also expect that net water availability will continue to decline as evaporative demand increases, consequentially increasing the likelihood of meteorological droughts from a supply and demand perspective. Overall, the attribution of changes in drought characteristics to anthropogenic climate change improves our understanding of historically observed trends and can contribute to our understanding of changes to come.

## Methods

To examine the overall impact of anthropogenic emissions on meteorological drought characteristics, we used monthly precipitation data from the Coupled Model Intercomparison Project Phase 6 (CMIP6) historical and historical natural-only model simulations (see Table 1 for the list of models used)^58,59^. The CMIP6 historical model simulations include all significant anthropogenic forcings (greenhouse gas emissions, anthropogenic aerosol emissions) and natural forcings (solar irradiance, stratospheric aerosols) in order to reflect historical observations^58^, while the historical natural-only simulations represent the historical period with natural forcings only. Comparing the differences between the two scenarios allows us to understand the impact of anthropogenic forcings on droughts. In addition, we used historical greenhouse-gas-only (abbreviated as GHG-only) simulations and historical anthropogenic-aerosol-only (abbreviated as AER-only) simulations to isolate the impacts of individual forcings on droughts^59^. To create multi-model ensembles of each scenario, we regridded the model output to a common 1 degree grid using nearest-neighbor interpolation and included all land area within 60°S and 60°N in our analysis.

**Table 1:** CMIP6 models used in the main text.

Modeling centre	Institute ID	Model name	Ensemble members
Beijing Climate Center, Beijing, China (BCC)	BCC	BCC-CSM2-MR	r1i1p1f1, r2i1p1f1, r3i1p1f1
Canadian Centre for Climate Modeling and Analysis	CCCma	CanESM5	r1i1p1f1, r2i1p1f1, r3i1p1f1
Centre National de Recherches Météorologiques/Centre Européen de Recherche et Formation Avancée en Calcul Scientifique	CNRM-CERFACS	CNRM-CM6-1	r1i1p1f2, r2i1p1f2, r4i1p1f2
LASG, Institute of Atmospheric Physics, Chinese Academy of Sciences and CESS, Tsinghua University	LASG-CESS	FGOALS-g3	r1i1p1f1, r2i1p1f1, r3i1p1f1
NASA Goddard Institute for Space Studies	NASA GISS	GISS-E2-1-G	r1i1p1f2, r2i1p1f2, r3i1p1f2
National Institute of Meteorological Research/Korea Meteorological Administration	MOHC	HadGEM3-GC31-LL	r1i1p1f3, r2i1p1f3, r3i1p1f3
Institut Pierre-Simon Laplace	IPSL	IPSL-CM6A-LR	r1i1p1f1, r2i1p1f1, r3i1p1f1
Atmosphere and Ocean Research Institute (The University of Tokyo), National Institute for Environmental Studies and Japan Agency for Marine-Earth Science and Technology	MIROC	MIROC6	r1i1p1f1, r2i1p1f1, r3i1p1f1
Meteorological Research Institute	MRI	MRI-ESM2-0	r1i1p1f1, r2i1p1f1, r3i1p1f1

Traditionally, drought indices are parametrically calculated through probability distribution fitting; however, creating comparable drought indices from different model simulations with this approach can be computationally challenging. Therefore, we use a non-parametric approach introduced in Farahmand and AghaKouchak (2015) to create our standardized indices to compare droughts under different modeled climate scenarios in a simple and consistent manner^60^. We first used the non-parametric standardized precipitation index (SPI) to represent the relative meteorological dryness of each pixel^60^. To quantify the features in the late 20^th^ century (1956–2005) and shifts between the late 19^th^ and 20^th^ centuries (1851–1900 and 1956–2005), we generated 6-month SPI values for each month in the 1851–2005 time series. The value corresponding to the month of June would include precipitation information from the 6-month period from January to June by first summing all monthly precipitation values. To create comparable SPI values across all scenarios (historical, historical natural-only, GHG-only, AER-only), we then ranked each month’s precipitation sum against the historical natural-only climatology of the corresponding pixel and month.

1 $$p\left( {x_i} \right) = \frac{{i - 0.44}}{{n + 0.12}}$$

Then, to create our index, we translated the empirical probabilities, *p*, from Eq. (1) into our standardized index (SI) with the standard normal distribution function with Eq. (2).

2 $${\mathrm{SI}} = \phi ^{ - 1}(p)$$

We defined drought frequency as the number of non-consecutive events below our defined SPI drought threshold of −1.5, which corresponds to the lower limit of a severe drought based on the U.S. Drought Monitor classification scheme^61^. We defined drought duration as the number of consecutive months associated with each drought event and drought intensity as the cumulative sum of SPI values associated with the drought months for each event. Maximum duration and maximum intensity refer to the maximum value for each pixel in each period. To test for the statistical significance of the shift in drought characteristics for each pixel, we implemented the one-sided sign test using the BSDA R package to test whether the median of the multi-model ensemble experienced a significant increase in each drought feature between the two time periods, controlling the false discovery rate with an *α*_FDR_ = 0.1^62–64^.

Using the median of the multi-model ensemble, we also constructed spatial probability distribution functions (PDFs) for each drought feature shift using a global aggregation approach. We aggregated all land pixels between 60°N and 60°S for each drought feature in historical and historical natural-only conditions to evaluate how the presence of anthropogenic forcing has impacted the distribution of each feature shift. We estimated the spatial PDFs by fitting kernel density estimates to our extracted data points^65^. We also conducted two-tailed two-sample *t*-tests on each drought feature to test for the statistical significance of the difference between the historical and historical natural-only distributions.

With the most recent 50 years of our model ensembles (1956–2005), we calculated probability ratios (PR) to demonstrate the recent impact of anthropogenic forcings on drought occurrences. The PR of each pixel is represented by a simple ratio:

3 $${\mathrm{PR}} = P_1/P_0$$

where *P*_0_ represents the probability of a drought occurring in historical natural-only conditions and *P*_1_ represents the probability of a drought occurring in anthropogenically forced conditions (GHG-only, AER-only, or all anthropogenic forcings)^18^.

Using regions denoted in the 5^th^ Intergovernmental Panel on Climate Change (IPCC) Assessment Report (IPCC regions), we also examined whether the bivariate distribution between drought duration and intensity was statistically different between the historical and historical natural-only scenarios^32^. We created each bivariate distribution from the total duration and monthly median intensity of each drought event detected from each CMIP6 model and implemented Fasano and Franceschini’s version of the 2-dimensional, 2-sample Kolmogorov–Smirnov test to test for statistical significance in each IPCC region^45,46^.

We also employed the standardized precipitation-evapotranspiration index (SPEI) to examine the additional impact of atmospheric demand on changes in drought features^30,66^. We first estimated monthly potential evapotranspiration (PET) with the FAO-56 Penman-Monteith equation (using a short reference crop with a height of 0.12 m) using the SPEI R package^67,68^. Our monthly-scale inputs for maximum and minimum temperature, wind speed, and cloud area fraction came from the same CMIP6 models used for the SPI analysis and 1 degree elevation data came from the Rand Corporation/Scripps Institution of Oceanography (accessed through the Cooperative Institute for Climate, Ocean, and Ecosystem Studies http://research.jisao.washington.edu/data_sets/elevation/). After deriving monthly PET values, we found the difference between precipitation and PET, *D*, for each month, *i*, with Eq. (4)^67^.

4 $$D_i = P_i - {\mathrm{PET}}_i$$

Following the SPI aggregation scheme, the *D* values were summed at the 6-month time scale and non-parametrically standardized using Eqs. (1) and (2) to create non-parametric SPEI datasets that correspond with the climate simulations^60^. We evaluated whether the median of the multi-model ensemble experienced a significant increase in drought frequency, duration, and intensity between 1851–1900 and 1956–2005, again controlling for the false discovery rate with an *α*_FDR_ = 0.1. In addition, we evaluated the probability ratios of SPEI-type droughts under historical, GHG-only, and AER-only climate conditions during 1956–2005.

## Supplementary information

Supplementary Information

## Data Availability

The CMIP6 data used in this study can be accessed online through the Earth System Grid Federation (ESGF) system. The local node used in this study is https://esgf-node.llnl.gov/search/cmip6/. The elevation data from the Rand Corporation / Scripps Institution of Oceanography can be accessed through the Cooperative Institute for Climate, Ocean, and Ecosystem Studies, http://research.jisao.washington.edu/data_sets/elevation/.

## Code availability

The base code used to generate the non-parametric SPI and SPEI indices can be found here: http://amir.eng.uci.edu/sdat.php and upon request, associated code used for the study can be provided by the corresponding author.

## References

[1] Wilhite DA, Svoboda MD, Hayes MJ (2007). Understanding the complex impacts of drought: a key to enhancing drought mitigation and preparedness. Water Resour. Manag..

[2] Vicente-Serrano SM (2013). Response of vegetation to drought time-scales across global land biomes. PNAS.

[3] Seneviratne, S. I. et al. *Managing the Risks of Extreme Events and Disasters to Advance Climate Change Adaptation* (eds Field, C. B., Barros, V., Stocker, T. F. & Dahe, Q.) (Cambridge Univ. Press, 2012).

[4] Sternberg T (2011). Regional drought has a global impact. Nature.

[5] Dai A (2011). Drought under global warming: a review. WIREs Clim. Chang..

[6] Tarroja B, Chiang F, AghaKouchak A, Samuelsen S (2018). Assessing future water resource constraints on thermally based renewable energy resources in California. Appl. Energy.

[7] Forrest K, Tarroja B, Chiang F, AghaKouchak A, Samuelsen S (2018). Assessing climate change impacts on California hydropower generation and ancillary services provision. Climatic Chang..

[8] Fischer, E. M., Seneviratne, S. I., Lüthi, D. & Schär, C. Contribution of land-atmosphere coupling to recent European summer heat waves. *Geophys. Res. Lett.* **34** 10.1029/2006GL029068 (2007).

[9] AghaKouchak A (2018). How do natural hazards cascade to cause disasters?. Nature.

[10] Spinoni J, Naumann G, Carrao H, Barbosa P, Vogt J (2014). World drought frequency, duration, and severity for 1951–2010. Int. J. Climatol..

[11] Nasrollahi N (2015). How well do CMIP5 climate simulations replicate historical trends and patterns of meteorological droughts?. Water Resour. Res..

[12] Spinoni J, Vogt JV, Naumann G, Barbosa P, Dosio A (2018). Will drought events become more frequent and severe in Europe?. Int. J. Climatol..

[13] Martin ER (2018). Future projections of global pluvial and drought event characteristics. Geophys. Res. Lett..

[14] Duffy PB, Brando P, Asner GP, Field CB (2015). Projections of future meteorological drought and wet periods in the Amazon. PNAS.

[15] Vicente‐Serrano SM (2020). Global characterization of hydrological and meteorological droughts under future climate change: the importance of timescales, vegetation-CO_2_ feedbacks and changes to distribution functions. Int. J. Climatol..

[16] Spinoni J (2020). Future global meteorological drought hot spots: a study based on CORDEX data. J. Clim..

[17] Touma D, Ashfaq M, Nayak MA, Kao S-C, Diffenbaugh NS (2015). A multi-model and multi-index evaluation of drought characteristics in the 21st century. J. Hydrol..

[18] Fischer EM, Knutti R (2015). Anthropogenic contribution to global occurrence of heavy-precipitation and high-temperature extremes. Nat. Clim. Chang..

[19] Easterling DR, Kunkel KE, Wehner MF, Sun L (2016). Detection and attribution of climate extremes in the observed record. Weather Clim. Extremes.

[20] Williams AP (2015). Contribution of anthropogenic warming to California drought during 2012–2014. Geophys. Res. Lett..

[21] Fischer EM, Knutti R (2014). Detection of spatially aggregated changes in temperature and precipitation extremes. Geophys. Res. Lett..

[22] Zhang X (2007). Detection of human influence on twentieth-century precipitation trends. Nature.

[23] Stott PA, Stone DA, Allen MR (2004). Human contribution to the European heatwave of 2003. Nature.

[24] Stott PA (2010). Detection and attribution of climate change: a regional perspective. Wiley Interdiscip. Rev..

[25] Hidalgo HG (2009). Detection and attribution of streamflow timing changes to climate change in the Western United States. J. Clim..

[26] Barnett TP (2008). Human-induced changes in the hydrology of the Western United States. Science.

[27] Wehner, M. F., Arnold, J. R., Knutson, T., Kunkel, K. E. & LeGrande, A. N. Ch. 8: Droughts, Floods, and Wildfires. Climate Science Special Report: Fourth National Climate Assessment, Volume I. https://science2017.globalchange.gov/chapter/8/ (2017) 10.7930/J0CJ8BNN.

[28] Marvel K (2019). Twentieth-century hydroclimate changes consistent with human influence. Nature.

[29] Bonfils CJW (2020). Human influence on joint changes in temperature, rainfall and continental aridity. Nat. Clim. Chang..

[30] Vicente-Serrano SM, Beguería S, López-Moreno JI (2009). A multiscalar drought index sensitive to global warming: the standardized precipitation evapotranspiration index. J. Clim..

[31] Sherwood S, Fu Q (2014). A drier future?. Science.

[32] IPCC. Climate Change 2013: The Physical Science Basis. Contribution of Working Group I to the Fifth Assessment Report of the Intergovernmental Panel on Climate Change. 10.1017/CBO9781107415324 (2013).

[33] Cook, B. I. et al. Twenty-First Century Drought Projections in the CMIP6 Forcing Scenarios. *Earth’s Future* **8**10.1029/2019EF001461 (2020).

[34] Held IM, Soden BJ (2006). Robust responses of the hydrological cycle to global warming. J. Clim..

[35] Seager R, Naik N, Vecchi GA (2010). Thermodynamic and dynamic mechanisms for large-scale changes in the hydrological cycle in response to global warming. J. Clim..

[36] Marvel K, Bonfils C (2013). Identifying external influences on global precipitation. PNAS.

[37] Wu P, Christidis N, Stott P (2013). Anthropogenic impact on Earth’s hydrological cycle. Nat. Clim. Chang..

[38] Mitchell JFB, Johns TC (1997). On modification of global warming by sulfate aerosols. J. Clim..

[39] Polson D, Bollasina M, Hegerl GC, Wilcox LJ (2014). Decreased monsoon precipitation in the northern hemisphere due to anthropogenic aerosols. Geophys. Res. Lett..

[40] Held IM, Delworth TL, Lu J, Findell Ku, Knutson T (2005). Simulation of Sahel drought in the 20th and 21st centuries. Proc. Natl Acad. Sci. USA.

[41] Bollasina MA, Ming Y, Ramaswamy V (2011). Anthropogenic aerosols and the weakening of the south Asian summer monsoon. Science.

[42] Biasutti, M. & Giannini, A. Robust Sahel drying in response to late 20th century forcings. *Geophys. Res. Lett.* **33**, 10.1029/2006GL026067 (2006).

[43] Booth BBB, Dunstone NJ, Halloran PR, Andrews T, Bellouin N (2012). Aerosols implicated as a prime driver of twentieth-century North Atlantic climate variability. Nature.

[44] Biasutti M (2019). Rainfall trends in the African Sahel: characteristics, processes, and causes. WIREs Clim. Chang..

[45] Fasano G, Franceschini A (1987). A multidimensional version of the Kolmogorov–Smirnov test. Mon. Not. R. Astron Soc..

[46] Brian Lau. 2-d Kolmorogov-Smirnov test, n-d energy test, Hotelling T^2 test. https://github.com/brian-lau/multdist (2016).

[47] Zhu Y, Zhang R-H, Sun J (2020). North Pacific upper-ocean cold temperature biases in CMIP6 Simulations and the role of regional vertical mixing. J. Clim..

[48] McKenna S, Santoso A, Gupta AS, Taschetto AS, Cai W (2020). Indian Ocean dipole in CMIP5 and CMIP6: characteristics, biases, and links to ENSO. Sci. Rep..

[49] Ropelewski CF, Halpert MS (1987). Global and regional scale precipitation patterns associated with the El Niño/southern oscillation. Mon. Wea. Rev..

[50] Tian, B. & Dong, X. The double-ITCZ bias in CMIP3, CMIP5, and CMIP6 models based on annual mean precipitation. *Geophys. Res. Lett.* **47**, e2020GL087232 (2020).

[51] Brown, J. R. et al. Comparison of past and future simulations of ENSO in CMIP5/PMIP3 and CMIP6/PMIP4 models. *Clim. Past Discuss.*10.5194/cp-2019-155 (2020).

[52] DeAngelis AM, Qu X, Hall A (2016). Importance of vegetation processes for model spread in the fast precipitation response to CO_2_ forcing. Geophys. Res. Lett..

[53] Burke EJ (2011). Understanding the sensitivity of different drought metrics to the drivers of drought under increased atmospheric CO_2_. J. Hydrometeor..

[54] Johnson F, Sharma A (2015). What are the impacts of bias correction on future drought projections?. J. Hydrol..

[55] Jiang Z, Sharma A, Johnson F (2019). Assessing the sensitivity of hydro-climatological change detection methods to model uncertainty and bias. Adv. Water Resour..

[56] Zhao C, Brissette F, Chen J, Martel J-L (2020). Frequency change of future extreme summer meteorological and hydrological droughts over North America. J. Hydrol..

[57] Joetzjer E (2013). Hydrologic benchmarking of meteorological drought indices at interannual to climate change timescales: a case study over the Amazon and Mississippi river basins. Hydrol. Earth Syst. Sci..

[58] Eyring V (2016). Overview of the Coupled Model Intercomparison Project Phase 6 (CMIP6) experimental design and organization. Geosci. Model Dev..

[59] Gillett NP (2016). The Detection and Attribution Model Intercomparison Project (DAMIP v1.0) contribution to CMIP6. Geosci. Model Dev..

[60] Farahmand A, AghaKouchak A (2015). A generalized framework for deriving nonparametric standardized drought indicators. Adv. Water Resour..

[61] Svoboda M (2002). The drought monitor. Bull. Am. Meteor. Soc..

[62] Arnholt, A. T. & Evans, B. BSDA: Basic Statistics and Data Analysis. (2017).

[63] Gibbons, J. D. & Chakraborti, S. *Nonparametric Statistical Inference: Revised and Expanded.* (CRC Press, 2014).

[64] Wilks DS (2016). “The Stippling Shows Statistically Significant Grid Points”: how research results are routinely overstated and overinterpreted, and what to do about it. Bull. Am. Meteor. Soc..

[65] Waskom, M. et al. seaborn: v0.7.0 (January 2016). 10.5281/zenodo.45133 (Zenodo, 2016).

[66] Beguería S, Vicente‐Serrano SM, Reig F, Latorre B (2014). Standardized precipitation evapotranspiration index (SPEI) revisited: parameter fitting, evapotranspiration models, tools, datasets and drought monitoring. Int. J. Climatol..

[67] Beguería, S. & Vicente-Serrano, S. M. SPEI: calculation of the standardised precipitation-evapotranspiration index (2017).

[68] Allen R, Smith M, Perrier A, Pereira LS (1994). An update for the definition of reference evapotranspiration. ICID Bull..

