# Supplementary material for: Evidence of anthropogenic impacts on global drought frequency, duration, and intensity
Source: Nat Commun. 2021 May 12;12:2754. doi: 10.1038/s41467-021-22314-w (PMC8115225; doi:10.1038/s41467-021-22314-w)
Supplement: Supplementary file 1 — Supplementary Information [file 41467_2021_22314_MOESM1_ESM.pdf]

Supplementary Information

Supplementary Table 1: **P-values from two-tailed two-sample t-test on SPI-based historical and historical natural-only spatial distributions from all land pixels between 60°N and 60°S.**

	p-value	t-value, DOF
Drought frequency	0	41.32, 14520
Maximum drought duration	3.69e-197	30.25, 20070
Maximum drought intensity	1.27e-266	-35.36, 18830

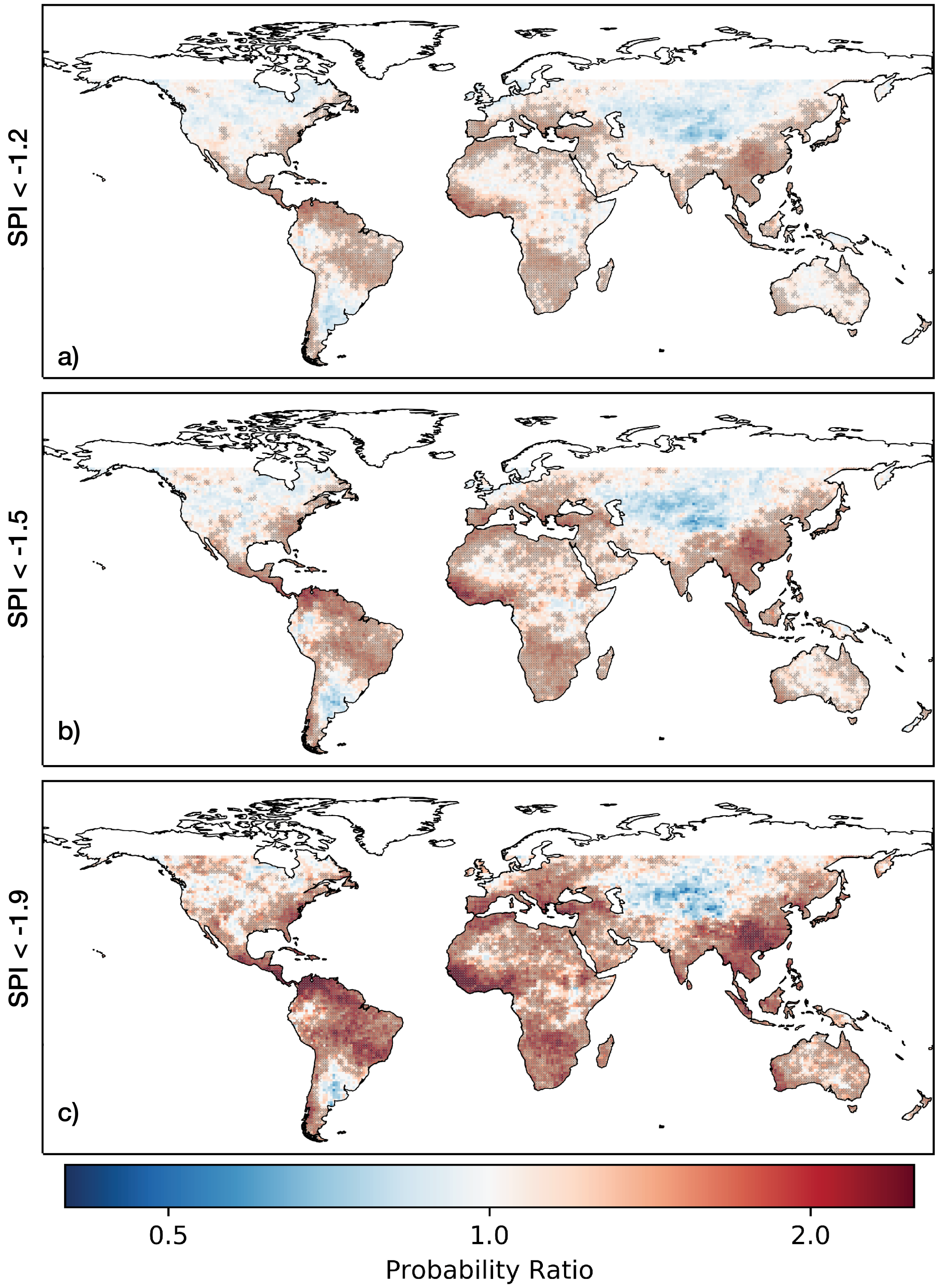

Supplementary Figure 1. **Probability Ratio (PR) of moderate, severe, and extreme drought events during the 1956-2005 model period. a)** Moderate, **b)** severe, and **c)** extreme drought events are defined as dips below 6-month SPI = -1.2, -1.5, and -1.9, respectively. Values above 1 indicate higher risks of drought events in historical conditions, while values below 1 indicate lower risks of drought events in historical conditions. Stippling over the grid cells indicates that the median of the model ensemble is statistically significantly greater than 1.

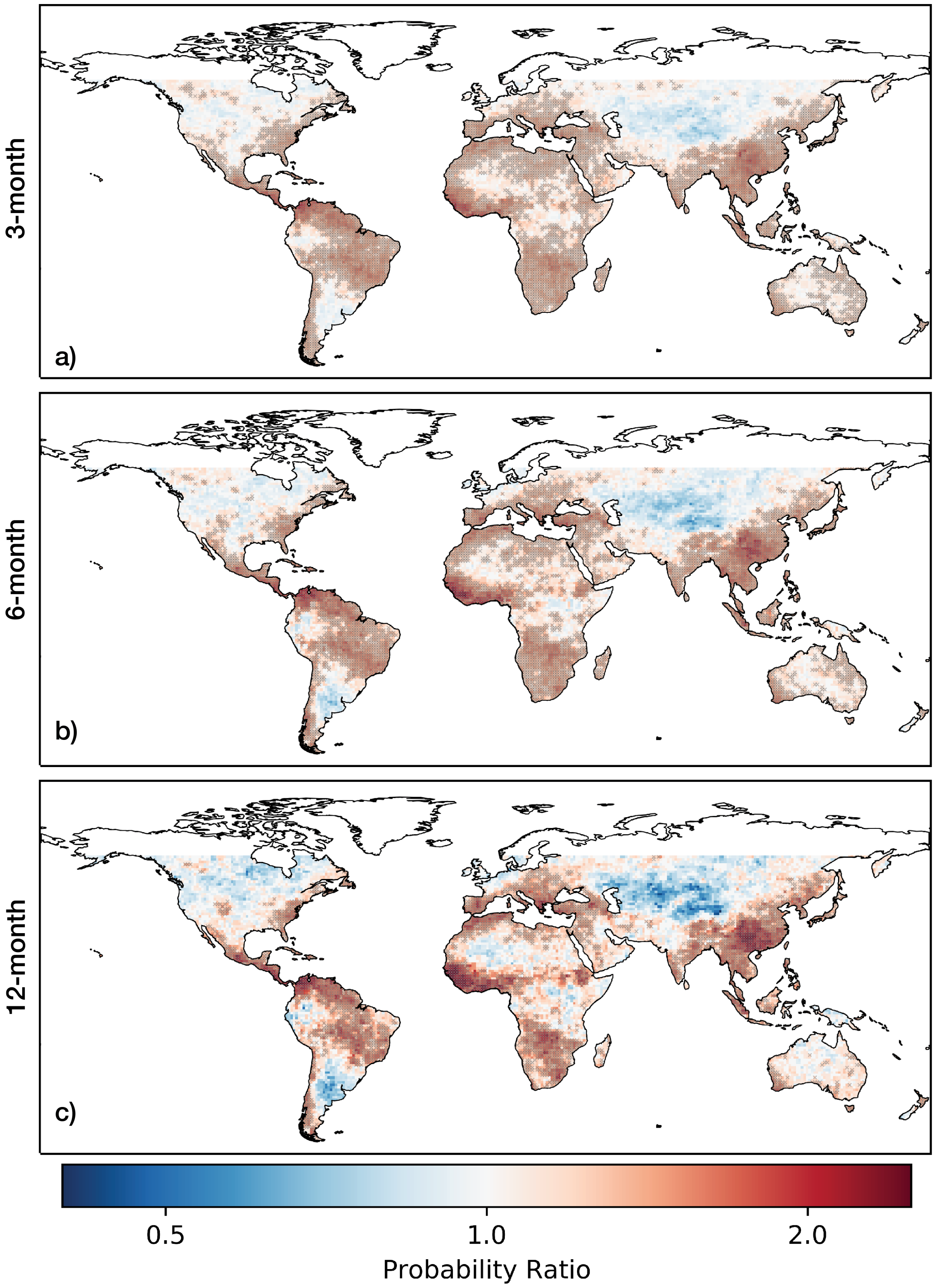

Supplementary Figure 2. **Probability ratios of drought occurrences generated from a) 3, b) 6, and c) 12 month windows.** Drought events are defined as non-consecutive events where the SPI index falls below -1.5. Values above 1 indicate higher risks of drought events in forced conditions, while values below 1 indicate lower risks of drought events. Stippling over the grid cells indicates that the median of the model ensemble is statistically significantly greater than 1.

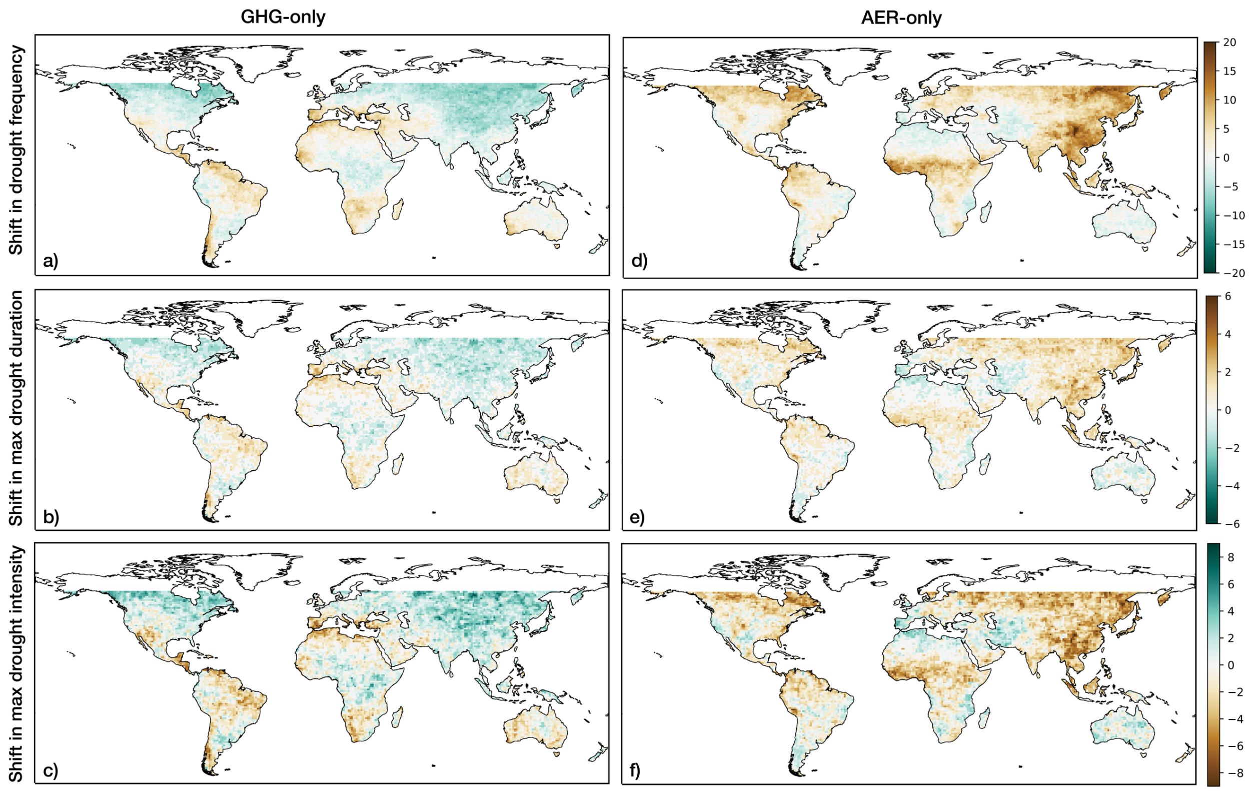

Supplementary Figure 3. **Shift in 6-month SPI < -1.5 drought features under greenhouse gas (GHG-only) versus aerosol (AER-only) climate scenarios. a)** Difference in drought frequency between 1956-2005 and 1851-1900 from the CMIP6 historical GHG-only multi-model ensemble median. **b)** Difference in maximum drought duration under historical GHG-only conditions. **c)** Difference in maximum drought intensity under historical GHG-only conditions. **d)** Difference in drought frequency under historical AER-only conditions. **e)** Difference in historical AER-only maximum drought duration. **f)** Difference in historical AER-only maximum drought intensity.

Supplementary Table 2: **2-sample 2-dimensional Kolmogorov-Smirnov test results for historical and historical natural-only bivariate distributions of drought event duration and median intensity.**

IPCC region	P-value	KS statistic
CNA	0.001308	0.092096
AMZ	2.19E-06	0.12542
CAM	2.59E-06	0.11044
CAS	0.02976	0.072495
CEU	0.00086527	0.0894
EAF	0.010236	0.078639
EAS	0.00011507	0.094082
ENA	5.23E-08	0.12895
MED	3.29E-11	0.15383
NAS	0.00054119	0.092618
NAU	0.0068893	0.079888
NEB	1.20E-05	0.11253
NEU	0.00016643	0.098426
SAF	0.00014447	0.09428
SAH	0.0007191	0.097385
SAS	1.76E-05	0.10631
SAU	0.00037035	0.097754
SEA	0.0019573	0.095545
SSA	7.23E-05	0.10636
TIB	0.08612	0.063483
WAF	6.23E-09	0.13645
WAS	5.45E-06	0.12114
WNA	0.00035011	0.098205
WSA	4.14E-08	0.13807

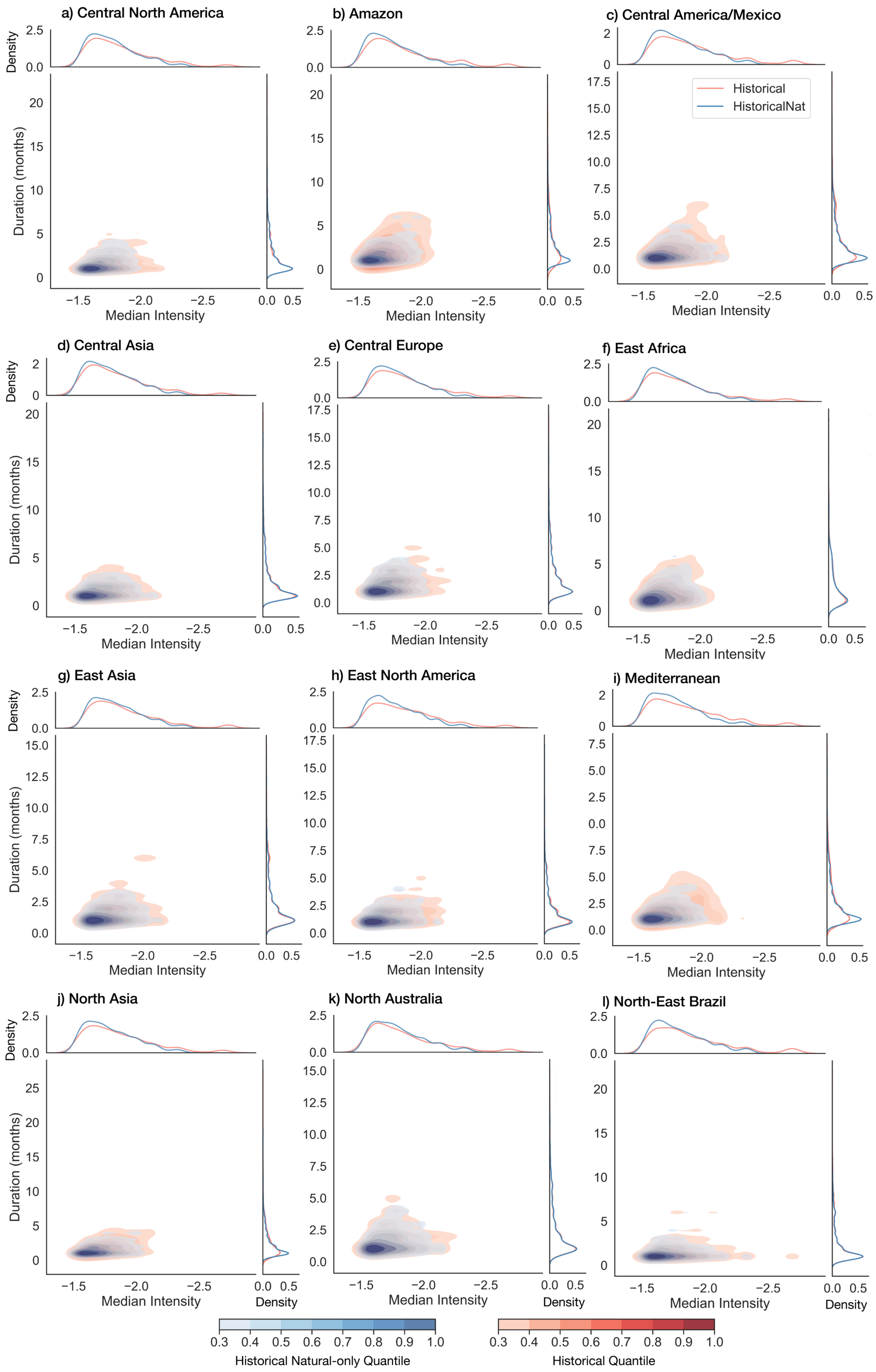

Supplementary Figure 4. **Drought event duration versus median intensity for IPCC regions.** The bivariate distributions in the subplots are based on SPI < -1.5 droughts identified from 6-month SPI data from all CMIP6 models. Univariate distributions of each characteristic are displayed on the outer axes.

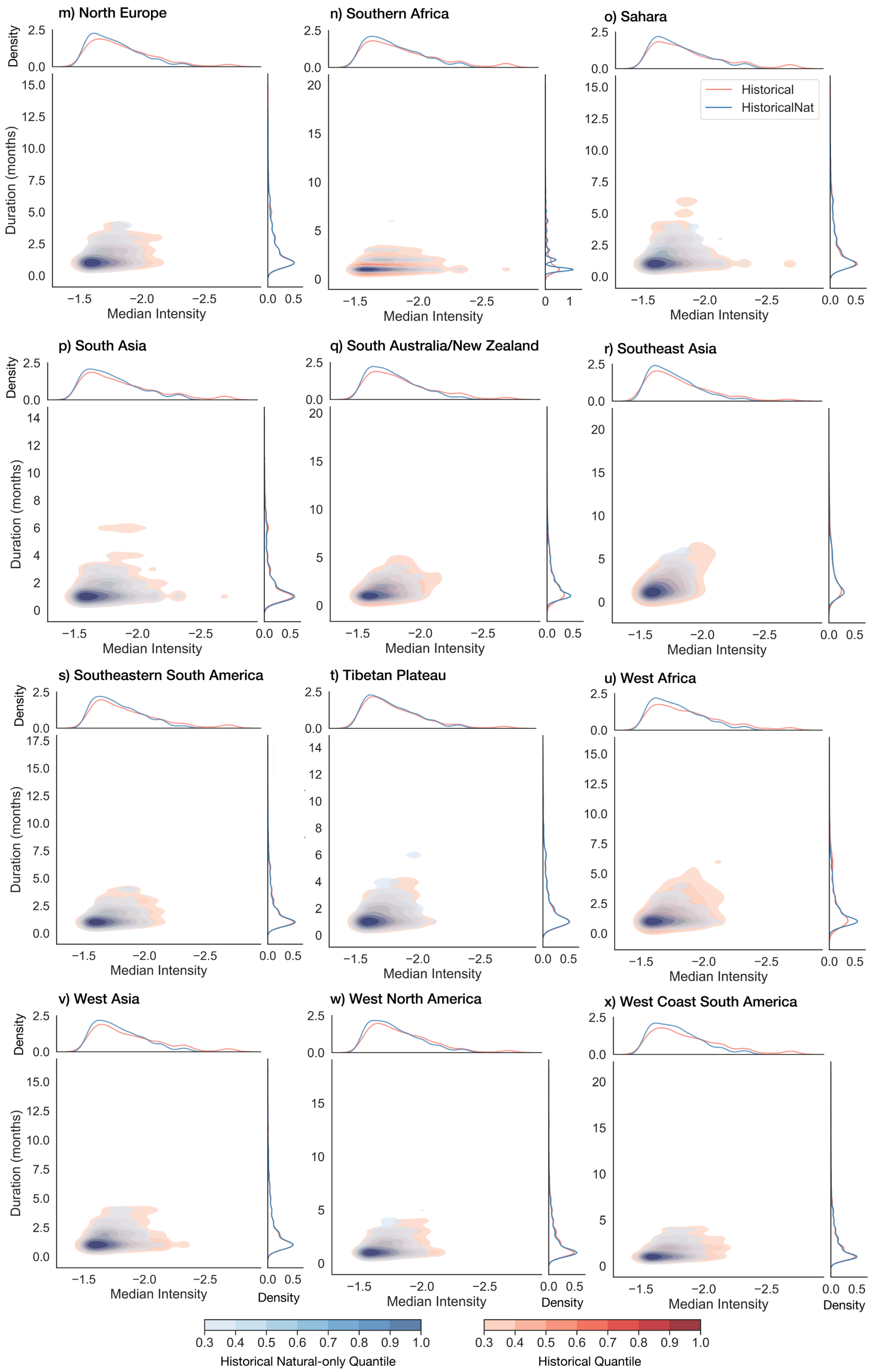

Supplementary Figure 4 (con't). **Drought event duration versus median intensity for IPCC regions.**

Supplementary Table 3: **P-values from two-tailed two-sample t-test on SPEI-based historical and historical natural-only spatial distributions from all land pixels between 60°N and 60°S.**

	p-value	t-value, DOF
Drought frequency	0	76.35, 12962
Maximum drought duration	0	64.69, 15635
Maximum drought intensity	0	-68.09, 14596

We previously examined the impact of anthropogenic forcing on shifts in 6-month SPI < -1.5 drought characteristics (1956-2005 relative to 1851-1900) and the likelihood of drought occurrences (1956-2005) using a multi-model ensemble of CMIP5 historical and historical natural-only simulations (list of models used in SM Table 4). From both SM Figures 5 and 6, we can see that the results of the study are broadly consistent when using CMIP5 or CMIP6 simulations. This was expected, given that CMIP5 and CMIP6 generally agree on regional wetting and drying patterns.

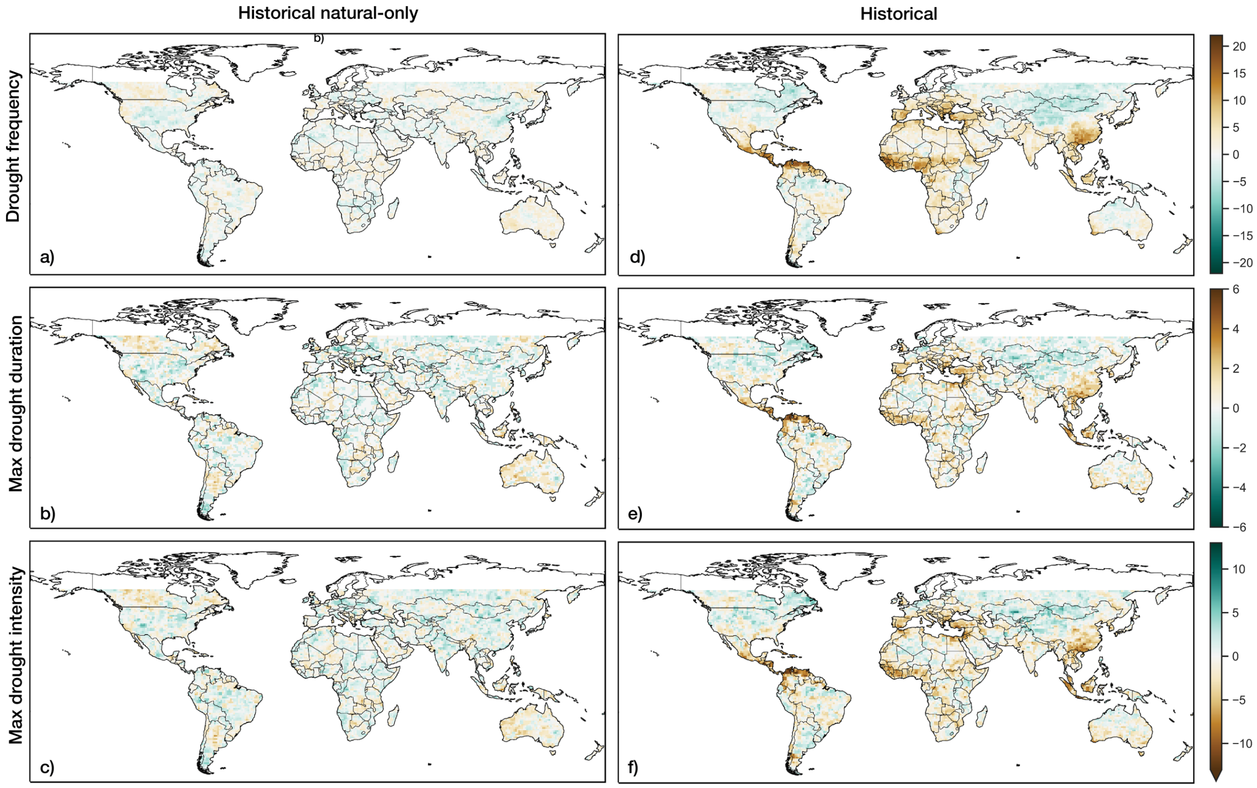

Supplementary Figure 5. **CMIP5 shift in 6-month SPI < -1.5 drought features under historical natural-only and historical climate scenarios. a)** Difference in drought frequency between 1956-2005 and 1851-1900 from the CMIP5 historical natural-only multi-model ensemble median. **b)** Difference in maximum drought duration under historical natural-only conditions**. c)** Difference in maximum drought intensity under historical natural-only conditions. **d)** Difference in drought frequency under historical conditions. **e)** Difference in historical maximum drought duration. **f)** Difference in historical maximum drought intensity.

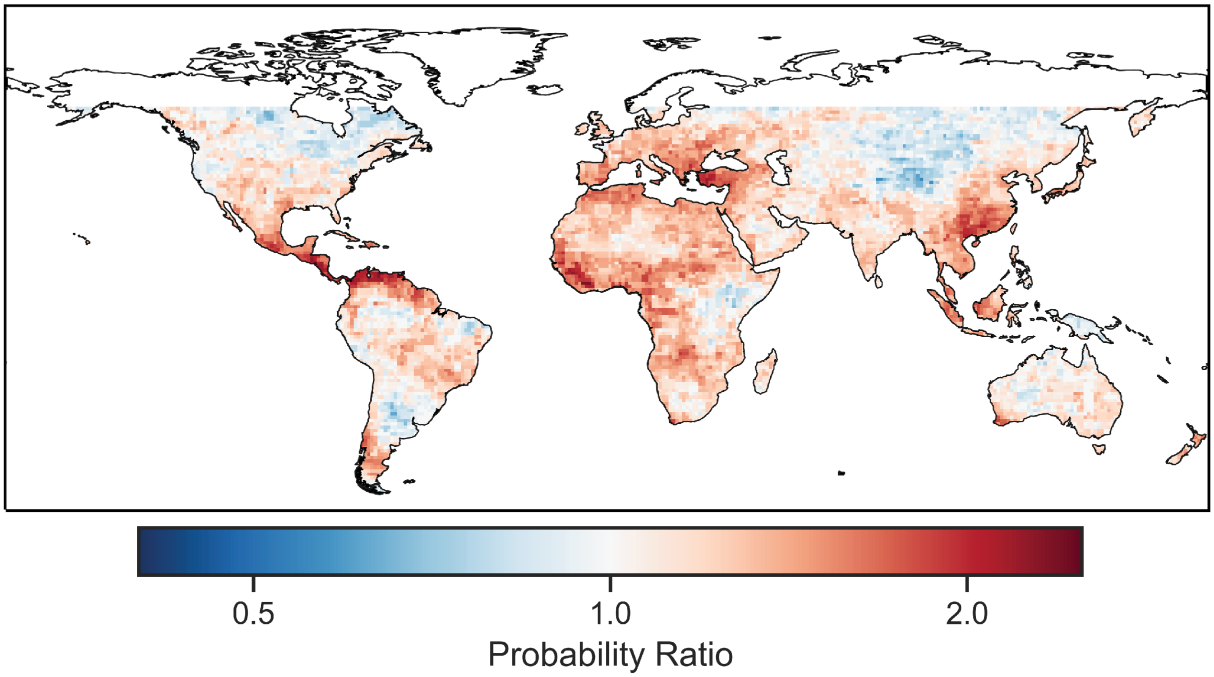

Supplementary Figure 6. **CMIP5 probability ratio of 6-month SPI < -1.5 drought occurrences under historical over historical natural-only conditions.** Each grid cell represents the probability ratio derived from the median of the model ensemble. Values above 1 indicate higher risks of drought events in anthropogenically forced conditions, while values below 1 indicate lower risks of drought events.

Supplementary Table 4: **CMIP5 models used in the supplementary materials**

Modeling Center	Institute ID	Model Name	Ensemble member
Commonwealth Scientific and Industrial Research Organization (CSIRO) and Bureau of Meteorology (BOM), Australia	CSIRO-BOM	ACCESS1.3	r1i1p1
Beijing Normal University (BNU)	BNU	BNU-ESM	r1i1p1
Canadian Centre for Climate Modeling and Analysis	CCCma	CanESM2	r1i1p1
National Center for Atmospheric Research	NCAR	CCSM4	r1i1p1
Centre National de Recherches Météorologiques / Centre Européen de Recherche et Formation Avancée en Calcul Scientifique	CNRM-CERFACS	CNRM-CM5	r1i1p1
Commonwealth Scientific and Industrial Research Organization in collaboration with Queensland Climate Change Centre of Excellence	CSIRO-QCCCE	CSIRO-Mk3-6-0	r1i1p1
LASG, Institute of Atmospheric Physics, Chinese Academy of Sciences and CESS, Tsinghua University	LASG-CESS	FGOALS-g2	r1i1p1
NASA Goddard Institute for Space Studies	NASA GISS	GISS-E2-H<br>GISS-E2-R	r1i1p1<br>r1i1p1
Institut Pierre-Simon Laplace	IPSL	IPSL-CM5A-LR<br>IPSL-CM5A-MR	r1i1p1<br>r1i1p1
Atmosphere and Ocean Research Institute (The University of Tokyo), National Institute for Environmental Studies and Japan Agency for Marine-Earth Science and Technology	MIROC	MIROC-ESM<br>MIROC-ESM-CHEM	r1i1p1<br>r1i1p1
Meteorological Research Institute	MRI	MRI-CGCM3	r1i1p1
Norwegian Climate Centre	NCC	NorESM1-M	r1i1p1
